# Supplementary material for: Exercise Training Enhances Platelet Mitochondrial Bioenergetics in Stroke Patients: A Randomized Controlled Trial
Source: J Clin Med. 2019 Dec 11;8(12):2186. doi: 10.3390/jcm8122186 (PMC6947198; doi:10.3390/jcm8122186)
Supplement: Supplementary file 1 [file jcm-08-02186-s001.pdf]

Supplementary Materials:

Figure S1: Flowchart of enrolled stroke patients during following-up. Inclusion and exclusion criteria listed in the figure were used to recruit eligible candidates. Recruited stroke patients were divided into two groups based on their treatments: TRP with (**TRP+ET**) and without ET (**TRP**). In addition to TRP, the TRP+ET group performed supervised in-hospital training on a bicycle ergometer (i.e., 50~60%VO_{2peak} for 30 min/day, 5 days/week for 4 weeks). The **TRP** group only engaged in traditional rehabilitation course for 4 weeks, as instructed by their rehabilitation physicians.

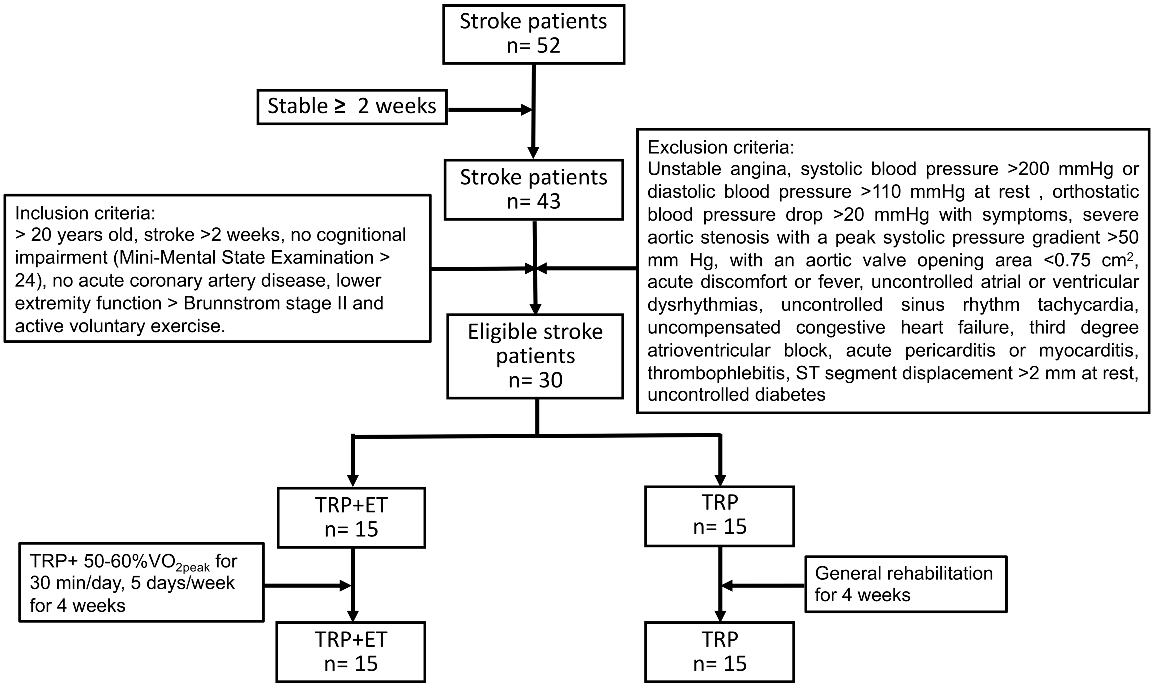

Figure S2: Graphs showing the reference protocols [(**A**) **RP1** and (**B**) **RP2**] of substrate-uncoupler-inhibitor titration (**SUIT**) and (**C**) convergent electron transfers at levels of the Q-junction [Complexes I and II (**CI** and **CII**), glycerophosphate dehydrogenase (**GpDH**), and electron-transferring flavoprotein (**ETF**)] in platelets. RP1 and RP2 are the SUIT experiments to measure mainly the capacities of mitochondrial oxidative phosphorylation (**OXPHOS**) and electron transport chain (**ETC**) in platelets, respectively. **LEAK**, uncoupling proton leakage; **ADP**, adenosine diphosphate; **P**, pyruvate; **M**, malate; **G**, glutamate; **S**, succinate; **Oct**, octanoyl-carnitine; **Gp**, glycerophosphate; **FCCP**, carbonyl cyanide-p-trifluoromethoxyphenylhydrazone; **Rot**, rotenone; **Ama**, antimycin A.

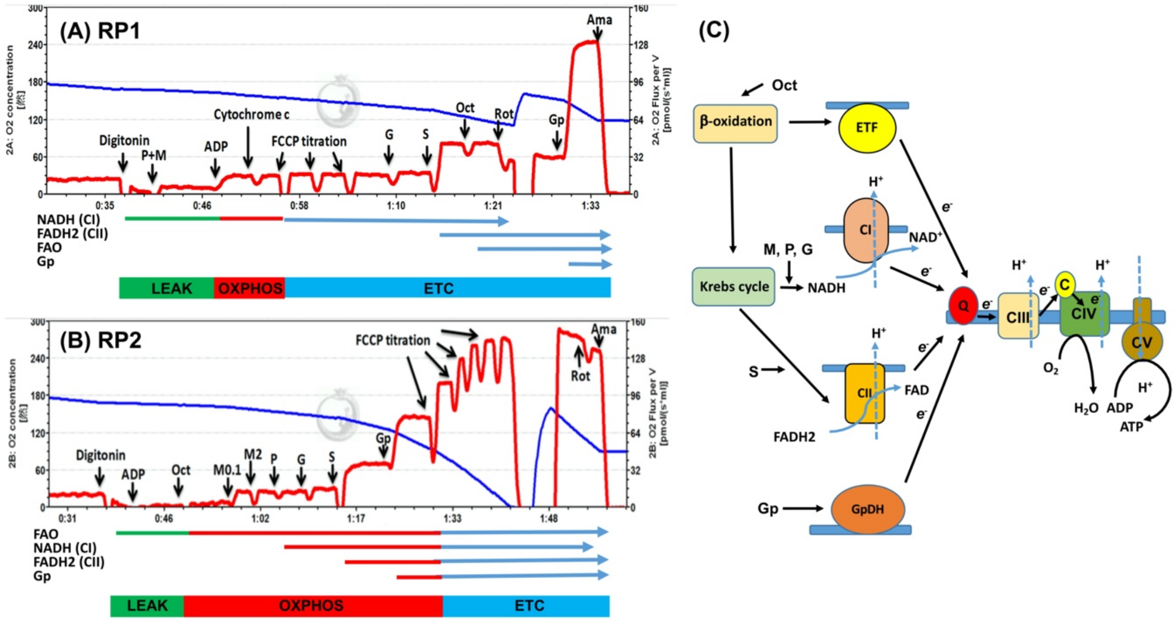

Table S1: Baseline demographics in hemorrhagic stroke patients.

		TRP+ET	TRP
Anthropometrics/Clinical Characteristics			
Gender	n (M/F)	3 (3/0)	3 (3/0)
Age	year	55.3±5.4	59.0±5.7
BMI	kg/m^2	23.7±1.6	28.3±1.6
Heart rate	bpm	69±3	71±5
Systolic blood pressure	mmHg	133±16	149±5
Diastolic blood pressure	mmHg	87±16	99±7
Stroke duration	month	29.0±12	26.2±14
Brunnstrom stage			
>Stage III	n (%)	3 (100)	3 (100)
Mini-Mental State Examination	score	28.3±1.7	29.0±0.8
Risk factors			
Smoking	n (%)	0 (0)	0 (0)
Hyperlipidemia	n (%)	1 (33)	1 (33)
Hypertension	n (%)	3 (100)	3 (100)
CVD	n (%)	1 (33)	1 (33)
Diabetes mellitus	n (%)	1 (33)	1 (33)
Medicines			
ASA	n (%)	0 (0)	0 (0)
HMG CoA reductase inhibitor	n (%)	1 (33)	1 (33)
β-blockers	n (%)	2 (67)	0 (0)
ACEI/ARB	n (%)	2 (67)	2 (67)

Values are mean ± SEM. ACEI/ARB, angiotensin converting enzyme inhibitor/angiotensin II receptor blocker; ASA, acetylsalicylic acid; BMI, body mass index; CVD, cardiovascular diseases; ET, exercise training; HMG CoA, 3-hydroxy-3-methyl- glutaryl coenzyme A; TRP, traditional rehabilitation program.

Table S2: Estimated power in measured parameters

Parameter	F-value	*p*-value	λ	Power
VO_{2peak}	53.434	< 0.0001	53.434	1.000
Max. ETC	52.242	<0.0001	52.242	1.000
ETC_{CI+CII}	21.160	< 0.0001	21.160	0.997
$ETC_{CI+CII+FAO}$	13.028	0.0012	13.028	0.952
ETC_{CII}	10.742	0.0028	10.742	0.902
ETC_{CII+Gp}	16.109	0.0004	16.109	0.983
Max. OXPHOS	19.226	0.0001	19.228	0.995

ETC_{CII}, electron transport chain of complex I in mitochondria; ETC_{CI+CII}, electron transport chain in complex I & II of mitochondria; ETC_{CII+Gp}, electron transport chain in complex II & glycerophosphate of mitochondria; $ETC_{CI+CII+FAO}$, electron transport chain of complex I, II, & fatty acid oxidation in mitochondria; Max. ETC, maximal electron transport chain capacity; Max. OXPHOS, maximal oxidative phosphorylation capacity; VO_{2peak}, peak exercise capacity.
